# Hyperactivity in male and female mice manifests differently following early, acute prenatal alcohol exposure and mild juvenile stress

**DOI:** 10.3389/fnbeh.2025.1501937

**Published:** 2025-03-18

**Authors:** Amy F. Pietrantonio, Raluca A. Urian, Daniel B. Hardy, Brian L. Allman, Katherine E. Willmore

**Affiliations:** ^1^Department of Anatomy and Cell Biology, Schulich School of Medicine and Dentistry, The University of Western Ontario, London, ON, Canada; ^2^Department of Physiology and Pharmacology, Schulich School of Medicine and Dentistry, The University of Western Ontario, London, ON, Canada; ^3^Department of Obstetrics and Gynecology, Schulich School of Medicine and Dentistry, The University of Western Ontario, London, ON, Canada; ^4^Children’s Health Research Institute, London, ON, Canada

**Keywords:** prenatal alcohol exposure, stress, hyperactivity, depression, sex differences, age effects, mice

## Abstract

**Introduction:**

Chronic prenatal alcohol exposure (PAE) and severe juvenile stress independently contribute to hyperactive and depressive behavioral phenotypes, with their combination exacerbating these effects. However, while chronic PAE and traumatic juvenile stress are well-studied, little is known about the impact of early, acute PAE and mild juvenile stress on hyperactivity and depression. This knowledge gap is clinically relevant, as these milder early-life insults are common in Western societies. Here, we provide the first investigation into the effects of early, acute PAE and juvenile sub-chronic, unpredictable, mild stress (SUMS)—both independently and in combination—on hyperactivity and depressive-like behaviors in mice throughout the lifespan.

**Methods:**

We assessed hyperactivity through movement-related measures (i.e., distance traveled, thigmotaxis, and rearing), whereas depressive-like behaviors were evaluated using the u-shaped two-choice field and forced swim tests. Behavioural testing was performed on equivalent numbers of male and female offspring and repeated at juvenile, adolescent, and adult timepoints to enable assessment of sex and age effects.

**Results:**

Neither early, acute PAE, juvenile SUMS, nor their combination induced depressive-like behaviors at any age; findings in contrast to the more severe chronic PAE and stress insults used in previous studies. However, these milder early-life insults did result in various hyperactivity phenotypes in both the male and female offspring. For example, juvenile SUMS had the strongest impact on hyperactive behaviors across both sexes, but only the adolescent females exhibited increased emotionality-associated activity. Moreover, early, acute PAE—both alone and in combination with juvenile SUMS significantly increased movement during adolescence and adulthood exclusively in male offspring.

**Discussion:**

Thus, our collective findings not only indicate that early, acute PAE and juvenile SUMS influence hyperactivity in a sex- and age-dependent manner, but also highlight that their influence on hyperactive and depressive phenotypes do not simply mirror those of the more severe early-life insults. Given the potential prevalence of early, acute alcohol exposure and juvenile stress in Western society, further research is warranted to fully understand their long-term behavioral consequences.

## 1 Introduction

The early-life insults of chronic prenatal alcohol exposure (PAE) and traumatic juvenile stress are known to independently lead to hyperactive (Cheng et al., 2018; Sanchez Vega et al., 2013; Sturman et al., 2021; Torres Muñoz and Franklin, 2022) and depressive behavioral phenotypes (Bake et al., 2021; Caldwell et al., 2008; Carneiro et al., 2005; Fryer et al., 2007; He et al., 2020; Iñiguez et al., 2014; Negele et al., 2015; O’Connor and Kasari, 2000; Slone and Redei, 2002; Steinhausen and Spohr, 1998). Moreover, it is well-established that the combination of chronic PAE and traumatic juvenile stress exacerbates these behavioral phenotypes as demonstrated through both clinical (Henry et al., 2007; Price et al., 2017) and preclinical studies (Alberry et al., 2021; Alberry and Singh, 2016; Comeau et al., 2015; Hellemans et al., 2008, 2010; Kambeitz et al., 2019; Lam et al., 2018a). In contrast, our understanding of the effects of less severe forms of these insults, including early, acute PAE and mild juvenile stress is limited, with even less known about their combined outcomes. In fact, to our knowledge, there are no studies that have investigated the combined effects of early, acute PAE and mild juvenile stress on hyperactive or depressive behaviors in postnatal life. Related to this gap in knowledge, mounting evidence from work focused on the prevalence of binge drinking, unplanned pregnancies, and exposure to mild childhood stress suggests that early, acute PAE and mild juvenile stress are insults commonly experienced in society (Canadian Centre on Substance Use and Addiction, 2019; Craig et al., 2018; Legault et al., 2021; Wozniak et al., 2019).

While limited in number, preclinical studies have demonstrated that exposure to even a single dose of alcohol during critical points in development, can result in lasting neurobehavioral deficits in offspring. These single-dose studies have largely focused on exposure between gestational days (GD) 7–9, a period in mouse development that corresponds to gastrulation and neurulation and that has been shown to be particularly susceptible to the toxic effects of alcohol (Dumas and Rabe, 1994; Endres et al., 2005; Schambra et al., 2015, 2016, 2017; Wieczorek et al., 2015). Collectively, these studies have demonstrated that early, acute PAE can disrupt learning, memory, sensorimotor, and cognitive development as well as cause sex-dependent differences in anxiety levels in mouse offspring (Dumas and Rabe, 1994; Endres et al., 2005; Schambra et al., 2015, 2016, 2017; Wieczorek et al., 2015). The disruptions caused by early, acute PAE are evident throughout the life course of offspring from the neonatal period to late adulthood with evidence that some of these outcomes interact with the effects of age of the offspring (Dumas and Rabe, 1994; Schambra et al., 2016). Moreover, brain imaging studies have demonstrated that PAE during this gestational window can cause structural anomalies in brain regions that are associated with regulating learning, memory and behavior (Godin et al., 2010; Lipinski et al., 2012). While it is yet to be experimentally demonstrated, these structural changes could underlie the neurobehavioral deficits observed following early, acute PAE. Similarly, juvenile, mild sub-chronic stress has been associated with emotional reactivity, depression, anxiety, memory deficits, and hyperactivity (Ducottet and Belzung, 2004, 2005; He et al., 2020; Sadler and Bailey, 2016; Ueno et al., 2018). Related to these behaviors, preclinical studies have demonstrated that juvenile stress can cause structural changes in brain regions involved in learning, memory, executive functioning, and emotional behavior (Bian et al., 2015; Tan et al., 2021; Ueno et al., 2018). Therefore, juvenile stress may alter behavior through structural alterations of the brain. Taken together, these studies indicate acute PAE during early gestation, and mild juvenile stress are sufficient on their own to cause lasting neurobehavioral deficits. Based on these previous findings, it is reasonable to predict that these relatively mild insults will likewise influence offspring hyperactive and depressive-like behaviors.

Guided by past studies, it is imperative that a preclinical investigation into the effects of early, acute PAE, mild juvenile stress, and their combination on hyperactive and depressive behaviors be designed in such a way as to assess for any differential sex and age effects, as well as for potential variability in the behavioral outcomes among individuals of the same treatment group. The rationale for this experimental design is derived from studies which demonstrate that chronic PAE and severe juvenile stress impact hyperactive and depressive behaviors in a sex-dependent manner depending on the behavioral outcome. Specifically, hyperactive behaviors are more prevalent in males than in females (Flannigan et al., 2023; Herman et al., 2008). In contrast, females demonstrate more severe depressive outcomes following these early-life insults than males (Famy et al., 1998; Flannigan et al., 2023; Fryer et al., 2007; O’Connor and Kasari, 2000; Sayal et al., 2007; Wei et al., 2021; Whitaker et al., 2021). These differential sex effects in behavioral outcomes have likewise been demonstrated in preclinical models of chronic PAE and severe juvenile stress, indicating that these sex effects are robust and can be modeled in the laboratory (Bake et al., 2021; Dunčko et al., 2001; Hellemans et al., 2010; Spivey et al., 2009). Moreover, these differential sex effects interact with age, with hyperactive behavior being most prevalent in juvenile males, whereas depression in females worsens with age (Bond and Di Giusto, 1977; Brault and Lacourse, 2012; Clark et al., 2004; Espinet et al., 2022; Famy et al., 1998; Flannigan et al., 2023; Herman et al., 2008; Peleg-Raibstein and Feldon, 2011; Ueno et al., 2018). Clinically, these sex and age effects could have important implications for screening and therapeutic approaches. Therefore, it is important to consider if these differential sex and age effects on depressive and hyperactive behaviors extend to offspring following early, acute PAE and mild juvenile stress.

Considering intra-group variability, past studies that have used preclinical models of early, acute PAE and juvenile sub-chronic unpredictable mild stress (SUMS) independently have reported that these milder insults can lead to variable outcomes among individuals. For example, studies in our lab and others have reported that mice exposed to early, acute PAE display highly variable craniofacial phenotypes ranging from no discernable effect to complete holoprosencephaly (Draghici et al., 2021; Godin et al., 2010; Sulik et al., 1981; Sulik and Johnston, 1983). Similarly, SUMS has been shown to result in a sizeable interindividual variability across numerous behavior domains including locomotion, depression, anxiety and spatial learning (Carere et al., 2001; Ducottet et al., 2004; Grootendorst et al., 2001; Ruis et al., 2001; Touyarot, 2004). Likewise, we expect that early, acute PAE and juvenile SUMS will lead to variable hyperactive and depressive outcomes. Therefore, when looking at the effects of these milder stressors on hyperactive and depressive behaviors in offspring across the lifespan, it is important to include measures of variation of outcomes.

The present study provides the first investigation of the effects of early, acute PAE, juvenile SUMS and their combination on hyperactive and depressive-like behaviors in mice. We investigated these behaviors in male and female offspring through juvenile, adolescent, and adult timepoints to allow us to determine whether the differential sex and age effects described in studies of chronic PAE, and in models of severe juvenile stress persist after exposure to early, acute PAE and juvenile SUMS. Multiple measures of both hyperactive and depressive-like behavior are assessed, allowing us to compare the effects of these early-life insults on different manifestations of each behavior. Additionally, our approach includes previously established assessments of variability to determine how behavioral profiles among individuals differ. Our alcohol dosing model represents a binge-like exposure in humans during the third week of gestation, a time that precedes pregnancy detection (Legault et al., 2021). Our juvenile stress protocol was chosen to reflect mild stressors similar in severity and relative length of exposure to those commonly experienced by children such as academic stress and bullying (Ducottet and Belzung, 2004; Goto and Toyoda, 2015). While chronic PAE and severe juvenile stress are known to cause hyperactive and depressive-like behaviors in offspring, the effects of early, acute PAE and juvenile SUMS on these outcomes remain unknown. The dearth of studies focused on the effects of these milder forms of both PAE and stress represents a significant, and clinically relevant gap in our knowledge, as the proportion of individuals exposed to these relatively mild insults is likely much higher than appreciated (Canadian Centre on Substance Use and Addiction, 2019; Craig et al., 2018; Legault et al., 2021; Wozniak et al., 2019). Our comprehensive investigation of behavioral profiles following early, acute PAE, mild juvenile stress, and their combination across age, between sexes, and among individual offspring, provide much needed information on these understudied insults.

## 2 Methods

### 2.1 Experimental groups

To determine the effects of early, acute PAE, juvenile SUMS, and their combination on offspring depressive and hyperactive behaviors, we used four experimental mouse groups. These groups included: vehicle control (referred hereafter as Vehicle, *n* = 28; 13 females, 15 males), an early, acute PAE group (referred hereafter as Ethanol, *n* = 30; 15 females, 15 males), a group exposed to juvenile SUMS (referred hereafter as Stress, *n* = 29; 15 females, 14 males), and a group exposed to both early, acute PAE and juvenile SUMS (referred hereafter as Double Hit, *n* = 34; 17 females and 17 males). The specific treatments used to create these four groups are described in detail below. Offspring from all experimental groups underwent a battery of behavioral tests (described below) as juveniles, adolescents, and adults to determine the effects of these stressors on hyperactive and depressive-like behaviors across the lifespan. Multiple litters (*n* = 4–8) were included in each group to account for potential litter effects and to provide sufficient offspring to investigate variation in outcomes (Supplementary Table S1). Overall, we found very few significant litter effects. Additionally, there were no consistent patterns in the distribution of significant litter effects, suggesting that offspring behavioral outcomes were not influenced by litter.

C57BL/6 mice (Charles River Laboratories, Quebec, Canada) were used to create all experimental groups. Mice were housed in standard cages and maintained at 22°C on a 12-h:12-h light:dark cycle, with access to food (2018 Teklad Global 18% Protein Diet, Harlan Laboratories, Indianapolis, IN, USA) and water *ad libitum*. All animal experiments were performed based on the approved Animal Use Protocol by the subcommittee of Canadian Council of Animal Care, The University of Western Ontario in accordance with the ARRIVE guidelines^1^ (Kilkenny et al., 2010).

### 2.2 Ethanol exposure

Following established methods (Almeida et al., 2020; Bertola et al., 2013; Livy et al., 2003), eight-week-old C57BL/6 nulliparous pregnant dams were administered a single dose of 31.5% v/v ethanol (2 mL/100 g of bodyweight) via oral gavage on GD 7.5 to produce the Ethanol and Double Hit offspring groups. This timing represents the second half of gastrulation in mice and similar binge-like exposures to ethanol at this timepoint have been shown to produce structural alterations to the brain and disrupted neurobehavioral outcomes in offspring (Godin et al., 2010; Lipinski et al., 2012; Wieczorek et al., 2015). To produce the Vehicle and Stress offspring groups, dams were administered distilled water via oral gavage at an equivalent volume on GD 7.5.

#### 2.2.1 Blood alcohol concentration

To characterize the blood alcohol concentration (BAC) following our dosing protocol, we constructed a BAC curve using a separate cohort of dams. Eight-week-old nulliparous female C57BL/6 mice (*n* = 4) were mated overnight. The selection of females for dosing was based on the presence of a vaginal plug following mating and/or weight gain of at least 1.0 g by GD 7.5 (Mader et al., 2009). Selected females were administered ethanol as described in *2.2 Ethanol Exposure* and blood was collected from the lateral saphenous vein at 30-, 60-, 90-, and 120-min timepoints post-gavage. We also collected blood from water-dosed mice to serve as a measurement of background BAC. Plasma was isolated by centrifugation and BAC was quantified using the Analox analyzer model GM7 MicroStat (Analox Instruments, Lunenburg, MA) (Supplementary Figure S1A). BAC was normalized by subtracting background (water-dosed mice BAC) and plotted against time (Chang et al., 2019).

### 2.3 Juvenile stress

Offspring were subjected to a SUMS regimen between postnatal days (PD) 28–55, following juvenile behavioral testing and prior to adolescent behavioral testing. The juvenile SUMS protocol was adapted from previous stress protocols by Ducottet et al. (2004), consisting of mild stressors such as damp bedding, no bedding, cage tilt (45°), wet cage (i.e., empty cage filled with 24°C water to a depth of 1 cm for 10 min), short-term altered light cycle (i.e., reversal of light/dark cycle and succession of light/dark cycle every 30 min), and social stress (i.e., introduction to the (empty) cage of another mouse) (Ducottet et al., 2004; Ducottet and Belzung, 2004). The duration of each stress event was short, ranging from 10 min to 4 h with the total stress duration for a given day ranging from 3–9 h. To achieve unpredictability, these stressors were pseudo-randomized, wherein each mouse in the Stress and Double Hit groups underwent the same stress protocol but the order of stress events was random, and the number of events ranged from one to three per day. Vehicle and Ethanol mice underwent daily handling. The stress schedule is outlined in full in Supplementary Table S2. At the end of the SUMS protocol, mice were left 1 day without any stressor prior to adolescent behavioral testing.

#### 2.3.1 Corticosterone measurement

As a measure of chronic stress, we characterized lifetime corticosterone (CORT) levels by collecting hair samples following the completion of behavioral testing. Briefly, hair samples were washed, ground, and weighed. CORT was extracted from the ground hair using methanol, evaporated under nitrogen and heat, and reconstituted in phosphate buffered saline (Greff et al., 2019). Hair CORT concentration was quantified by an established enzyme-linked immunosorbent assay (ELISA) technique (Greff et al., 2019) and expressed as nanogram/gram (ng/g) of hair mass (Supplementary Figure S1B).

### 2.4 Behavioral testing

Offspring underwent behavioral testing to assess and compare activity levels and depressive-like phenotypes among experimental groups. Behavioral testing occurred at juvenile (PD 22–26), adolescent (PD 57–61), and adult (PD 120–124) timepoints. All behavioral testing occurred between 9 a.m. and 6 p.m., was recorded on ANY-maze video tracking software, and was assessed while blinded to the experimental group. On the final day of behavioral testing, adolescent and adult female offspring underwent estrous cycle staging as cycle stage and sex hormones can impact female behavior (Chari et al., 2020; Lovick and Zangrossi, 2021). Using a three-way ANOVA with ethanol, stress, and estrous cycle stage as main effects, we found that estrous cycle stage did not impact female behavioral outcomes.

#### 2.4.1 Hyperactivity in the U-shaped two-choice field

**Figure 1:**
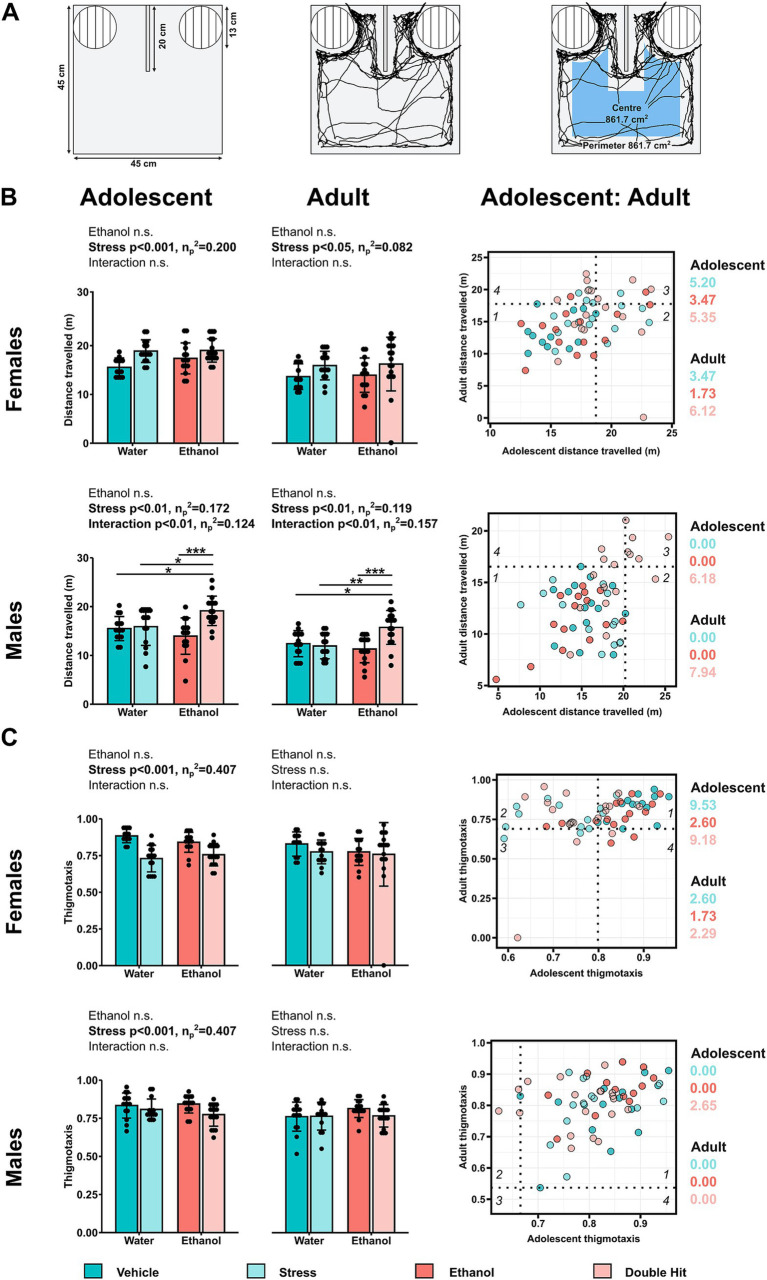
Prenatal alcohol exposure, juvenile stress, and their combination increase horizontal activity in an age- and sex-dependent manner during the habituation stage of the U-shaped two-choice field (U-field) test. **(A)** The unanimated U-field (left), representative trajectories of a subject mouse denoted by the black tracing for distance traveled (middle) and thigmotaxis (right) (created with BioRender.com). **(B)** Distance traveled in meters (m) and **(C)** thigmotaxis are shown for adolescent (left) and adult (middle) offspring from Vehicle (*n* = 28, 13 females, 15 males; 7 litters), Stress (*n* = 29, 15 females, 14 males; 4 litters), Ethanol (*n* = 30, 15 females, 15 males; 4 litters), and Double Hit (*n* = 34, 17 females, 17 males; 5 litters) experimental groups. Means for each group are represented by bars ± standard deviation. Data were compared using two-way ANOVAs (ethanol, stress), followed by Tukey’s *post hoc* analyses where appropriate. *p < 0.05, **p < 0.01, ***p < 0.001, n.s. = not significant. One-dimensional matrices (right) show horizontal activities at adolescent and adult timepoints for all offspring. The dotted black lines represent the highest Vehicle distance traveled **(B)** and lowest Vehicle thigmotaxis **(C)** at adolescent (vertical) and adult (horizontal) timepoints as determined by the highest/lowest 5th percentile. Quadrants 1 through 4 represent the following: (1) within Vehicle range at adolescent and adult timepoints; (2) outside the Vehicle range at adolescent, but within Vehicle range at adult timepoints; (3) outside the Vehicle range at both adolescent and adult timepoints; and (4) within Vehicle range at adolescent but outside at the adult timepoint. Relative risk of developing abnormal behavioural outcomes is shown on the right of the one-dimensional matrices.

Hyperactivity is characterized by excessive movement and restlessness (Magnus et al., 2023; Posner et al., 2020; Wilens and Spencer, 2010). In mice, hyperactivity can be measured through horizontal and vertical activities, where generally, increases in these activities are associated with hyperactivity (Gould et al., 2009; Kraeuter et al., 2019; Seibenhener and Wooten, 2015; Tatem et al., 2014). In the present study, four measures of offspring activity levels were assessed during the 5-min habituation period for the u-shaped two-choice field (U-field) test, including two horizontal activities and two vertical activities (Figure 1A).

Briefly, the subject mouse freely explored the unanimated U-field for 5 min, during which time mouse activity was recorded using ANY-maze video tracking software. Our horizontal activities included total distance traveled in meters (i.e., how much the mouse moved, Figure 1A) and thigmotaxis, a metric of perimeter preference (i.e., where the mouse moved, Figure 1A) (Cheng et al., 2018; Park et al., 2014; Sanchez Vega et al., 2013; Sturman et al., 2018). To determine thigmotaxis, the field was conceptually separated into a central area and a peripheral area, each comprised of 50% of the total surface area. The tracking software allowed us to measure total distance traveled and to calculate thigmotaxis as $\frac{\mathrm{time in perimeter}}{\mathrm{time in perimeter}+\mathrm{time in center}}$ . Increased distance traveled is associated with hyperactivity (Gould et al., 2009; Kraeuter et al., 2019). Contrastingly, decreased thigmotaxis (i.e., increased time in the center of the field) is associated with hyperactivity, though it should be noted that this parameter also provides information on emotional behavior (Sturman et al., 2018, 2021; Torres Muñoz and Franklin, 2022). Our vertical activities included supported rearing (i.e., rearing with forepaws contacting another surface), unsupported rearing (i.e., rearing without forepaws contacting another surface), and total rearing (i.e., supported and unsupported rearing summed together). We distinguished between supported and unsupported rearing as they provide us with different information; supported rearing is related to locomotion whereas similarly to thigmotaxis, unsupported rearing is related to both locomotion and emotional behavior (Katz et al., 1981; Seibenhener and Wooten, 2015; Sturman et al., 2018, 2021). These measures were recorded as the time spent engaged in these rearing activities in seconds, where increased rearing is associated with hyperactivity (Gould et al., 2009; Zhuang et al., 2001).

#### 2.4.2 Sociability in the U-shaped two-choice field

Given that depression is not a single phenotype, but rather a complex profile with variable symptoms, we measured depressive-like behavior with different tests. Depression is a mood disorder characterized by persistent sadness and loss of interest in previously enjoyable activities (Chand and Arif, 2023; Tan et al., 2021). In mice, social isolation can be measured as sociability (the tendency to seek social interaction), and past studies have suggested that reduced sociability is a characteristic of depression (Kaidanovich-Beilin et al., 2011; Planchez et al., 2019). Moreover, despair can be measured as psychomotor withdrawal or immobility, in which increased immobility is associated with depression (Planchez et al., 2019; Porsolt et al., 1977).

**Figure 2:**
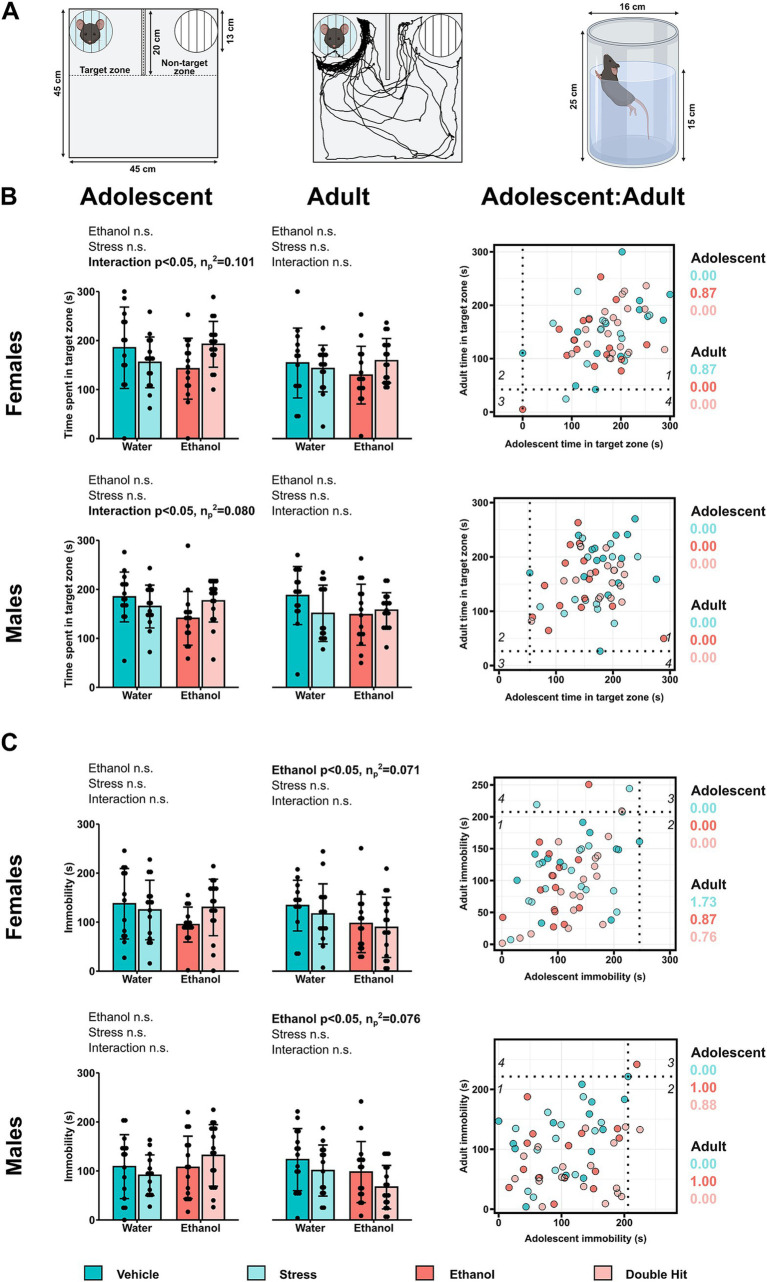
Prenatal alcohol exposure, juvenile stress, and their combination do not lead to depressive-like behaviors and perhaps induce an anti-depressant effect. **(A)** The animated U-shaped two-choice field (U-field); the quadrant in which the social target mouse was placed (“animated cage”) represents the “Target zone,” whereas the quadrant with the “unanimated cage” is the “Non-target zone” (left). Representative trajectory of a subject mouse denoted by the black tracing in the U-field in the context of non-mate target mouse vs. unanimated cage (middle). The forced swim test (FST) apparatus (right) (created with BioRender.com). **(B)** Time spent in the target zone in the U-field test and **(C)** time spent immobile in the FST is shown for adolescent and adult offspring from Vehicle (*n* = 28, 13 females, 15 males; 7 litters), Stress (*n* = 29, 15 females, 14 males; 4 litters), Ethanol (*n* = 30, 15 females, 15 males; 4 litters), and Double Hit (*n* = 34, 17 females, 17 males; 5 litters) experimental groups. Means for each group are represented by bars ± standard deviation. Data were compared using two-way ANOVAs (ethanol, stress). n.s. = not significant. One-dimensional matrices (right) show horizontal activities at adolescent and adult timepoints for all offspring. The dotted black lines represent the lowest Vehicle time in target zone **(B)** and highest Vehicle immobility **(C)** at adolescent (vertical) and adult (horizontal) timepoints as determined by the highest/lowest 5th percentile. Quadrants 1 through 4 represent the following: (1) within Vehicle range at adolescent and adult timepoints; (2) outside the Vehicle range at adolescent, but within Vehicle range at adult timepoints; (3) outside the Vehicle range at both adolescent and adult timepoints; and (4) within Vehicle range at adolescent but outside at the adult timepoint. Relative risk of developing abnormal behavioural outcomes is shown on the right of the one-dimensional matrices.

In the present study, the U-field test was used to investigate sociability. Following established methods (Lee et al., 2018; Park et al., 2014; Seo et al., 2012), offspring habituated to the testing room for a minimum of 20 min prior to testing. The test began with a habituation stage, wherein each subject mouse freely explored the unanimated U-field for 5 min. Following habituation, the subject mouse was returned to its home cage for 2 min, during which time an unfamiliar social target mouse (ensuring same sex, age, and strain as the subject mouse) was placed in a cage in quadrant 1 (target zone) while the cage in quadrant 3 remained empty (non-target zone) (Figure 2A). The subject mouse was then returned to the U-field to explore for 5 min for the sociability task. To quantify sociability, we measured the time spent in the target zone (Figure 2A), where decreased time spent in the target zone is associated with reduced sociability and thus, a depressive-like phenotype (Lee et al., 2018; Park et al., 2014; Seo et al., 2012).

#### 2.4.3 Forced swim test

In the present study, the forced swim test (FST) was used to investigate psychomotor withdrawal. The FST apparatus consists of a glass, cylindrical beaker (16 cm (D) × 25 cm (H)), filled to a height of 15 cm with 24°C ± 1°C water (Figure 2A). In this test, mice were placed in the water and the amount of time spent immobile was measured (Figure 2A). Mice were left in the beaker for 6 min, however, given that most mice are very active at the beginning of the test, time immobile was only measured during the last 5 min (Can et al., 2012; Park et al., 2014; Porsolt et al., 1977; Seo et al., 2012; Wieczorek et al., 2015). Increased time spent immobile is associated with increased behavioral despair and therefore, an increased depressive-like phenotype (Can et al., 2012; Porsolt et al., 1977).

### 2.5 Data analysis

As the juvenile testing period occurred before the stress exposure (PD 28–55), juvenile behavioral measures were analyzed using two-way ANOVAs with ethanol and sex as factors to determine the effect of ethanol alone on behavioral outcomes (distance traveled, thigmotaxis, rearing, sociability, immobility). For adolescent and adult data, behavioral measures were first analyzed using four-way mixed-model ANOVAs with ethanol (between comparisons), stress (between comparisons), sex (between comparisons), and age (within comparisons) as factors (Supplementary Table S3) which uncovered both significant age and sex effects. Behavioral measures were then analyzed using three-way ANOVAs with ethanol, stress, and sex as factors for ages separately (Supplementary Tables S4, S5). Given consistent significant sex effects, the results below arise from two-way ANOVAs with ethanol and stress as factors for ages and sexes separately. Statistical significance was defined as *p* < 0.05 and Tukey’s *post hoc* testing was performed as appropriate. Effect size was calculated using partial eta squared (η_p_^2^) analyses, and interpreted as ≤0.05 = small effect, 0.06–0.13 = medium effect, and ≥0.14 = large effect (Lakens, 2013).

**Figure 3:**
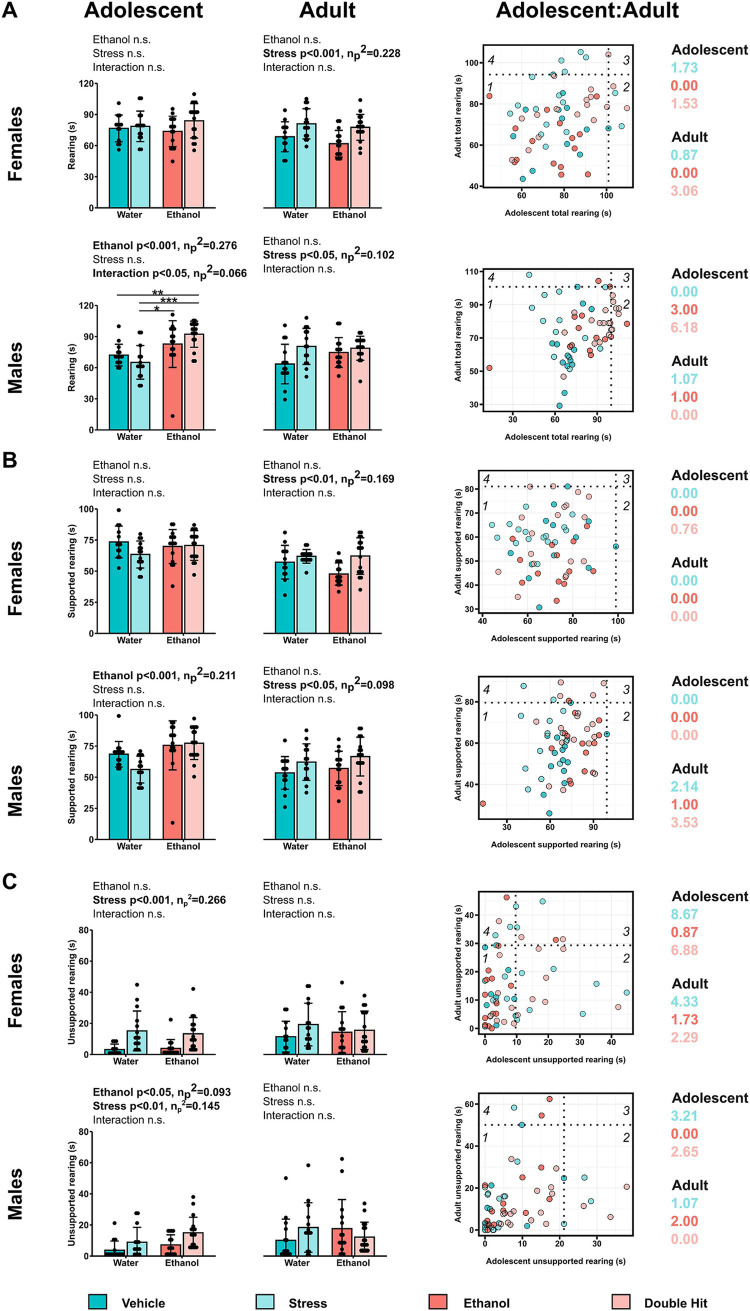
Prenatal alcohol exposure affects rearing behaviors in adolescent offspring, whereas adult rearing is affected by juvenile stress. Time in seconds spent **(A)** rearing in total, **(B)** in supported rearing, and **(C)** in unsupported rearing postures during the habituation stage of the U-shaped two-choice field (U-field) test are shown for adolescent and adult offspring from Vehicle (*n* = 28, 13 females, 15 males; 7 litters), Stress (*n* = 29, 15 females, 14 males; 4 litters), Ethanol (*n* = 30, 15 females, 15 males; 4 litters), and Double Hit (*n* = 34, 17 females, 17 males; 5 litters) experimental groups. Means for each group are represented by bars ± standard deviation. Data were compared using two-way ANOVAs (ethanol, stress), followed by Tukey’s *post hoc* analyses where appropriate. *p < 0.05, **p < 0.01, ***p < 0.001, n.s. = not significant. One-dimensional matrices (right) show vertical activities at adolescent and adult timepoints for all offspring. The dotted black lines represent the highest Vehicle total rearing **(A)**, supported rearing **(B)**, and unsupported rearing **(C)** at adolescent (vertical) and adult (horizontal) timepoints as determined by the highest 5th percentile. Quadrants 1 through 4 represent the following: (1) within Vehicle range at adolescent and adult timepoints; (2) outside the Vehicle range at adolescent, but within Vehicle range at adult timepoints; (3) outside the Vehicle range at both adolescent and adult timepoints; and (4) within Vehicle range at adolescent but outside at the adult timepoint. Relative risk of developing abnormal behavioural outcomes is shown on the right of the one-dimensional matrices.

Next, using an established method, we constructed one-dimensional matrices to investigate the effect of treatment for each behavioral measure across age (Figures 1–3). Raw behavioral measures were plotted, and we classified a given behavioral measure as “normal” if they were within the 5–95% percentile of the Vehicle group (Graeca and Kulesza, 2024). We used this classification to compare the distribution of normal/abnormal behavioral outcomes across experimental groups and age and to calculate relative risk (RR) of developing abnormal behavioral outcomes (Graeca and Kulesza, 2024). To interpret the one-dimensional matrices, the dotted black lines represent the highest/lowest Vehicle behavior at adolescent (vertical) and adult (horizontal) timepoints as determined by the highest/lowest 5th percentile. Quadrants 1 through 4 represent the following: (1) within Vehicle range at both adolescent and adult ages; (2) outside the Vehicle range as an adolescent, but within Vehicle range as an adult; (3) outside the Vehicle range at both adolescent and adult ages; and (4) within Vehicle range at adolescent but outside as an adult.

**Figure 4:**
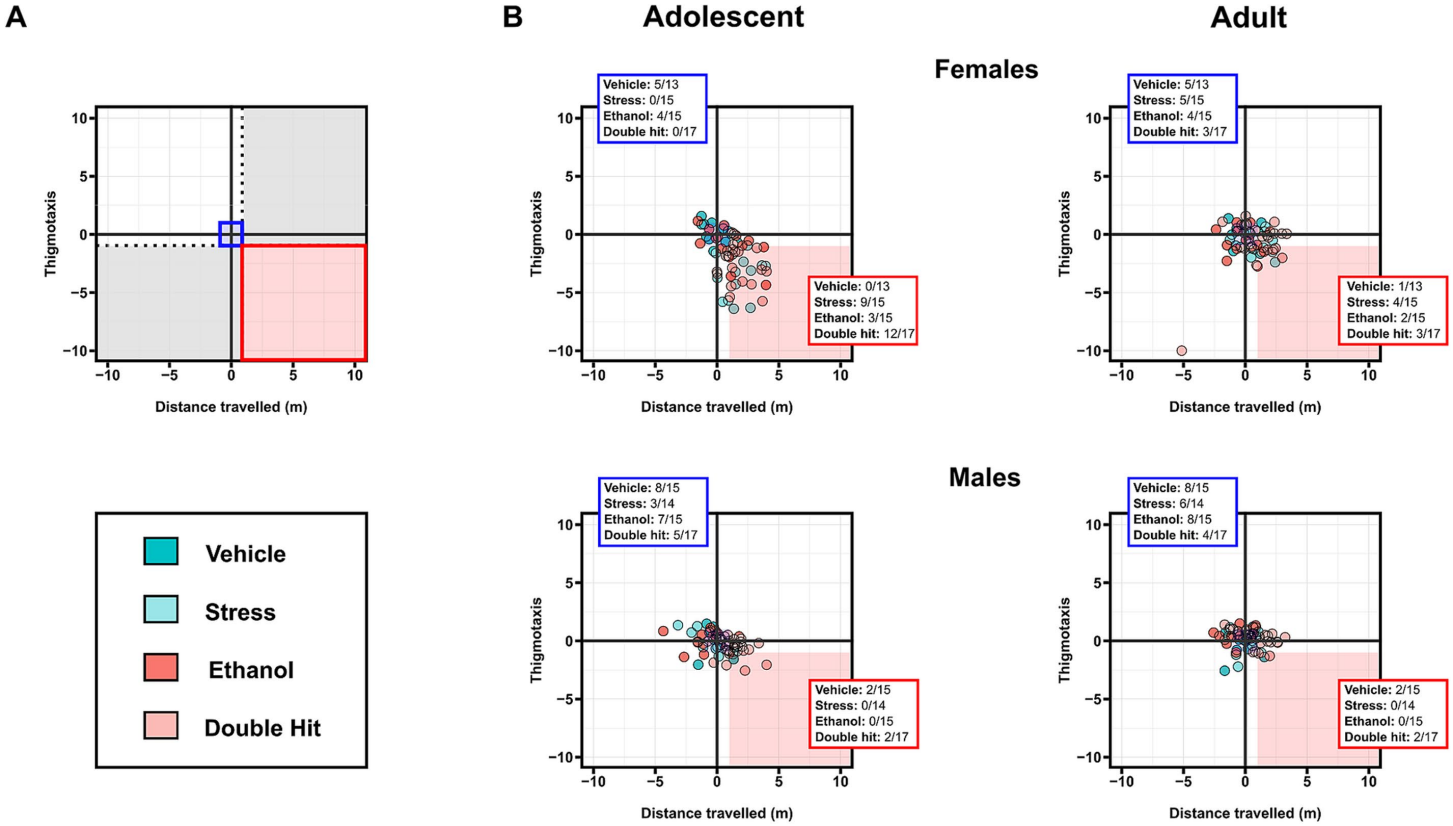
Prenatal alcohol exposure, juvenile stress, and their combination increases susceptibility to combined deficits in distance traveled and thigmotaxis in adolescent female offspring. **(A)** Representative two-dimensional matrix. The Vehicle group mean ± one standard deviation is represented by the blue-shaded box in the center of the matrix. The dotted black lines separate individuals who display depressive-like behaviors in only one measure, defined by being outside one standard deviation of the Vehicle group mean in that measure (areas shaded in gray). The blue-shaded box represents the Vehicle group mean ± one standard deviation. The red-shaded box represents individuals who display high distance traveled and low thigmotaxis, defined by being outside one standard deviation of the Vehicle group mean in both measures. Two-dimensional matrices of distance traveled x thigmotaxis for adolescent **(B)** and adult **(C)** offspring from Vehicle (*n* = 28, 13 females, 15 males; 7 litters), Stress (*n* = 29, 15 females, 14 males; 4 litters), Ethanol (*n* = 30, 15 females, 15 males; 4 litters), and Double Hit (*n* = 34, 17 females, 17 males; 5 litters) experimental groups. The proportion of individuals that fall within the red-shaded box is denoted in the red box in the bottom right corner of the respective matrix. The proportion of individuals that fall within the blue-shaded box is denoted in the blue box in the top left corner of the respective matrix.

**Figure 5:**
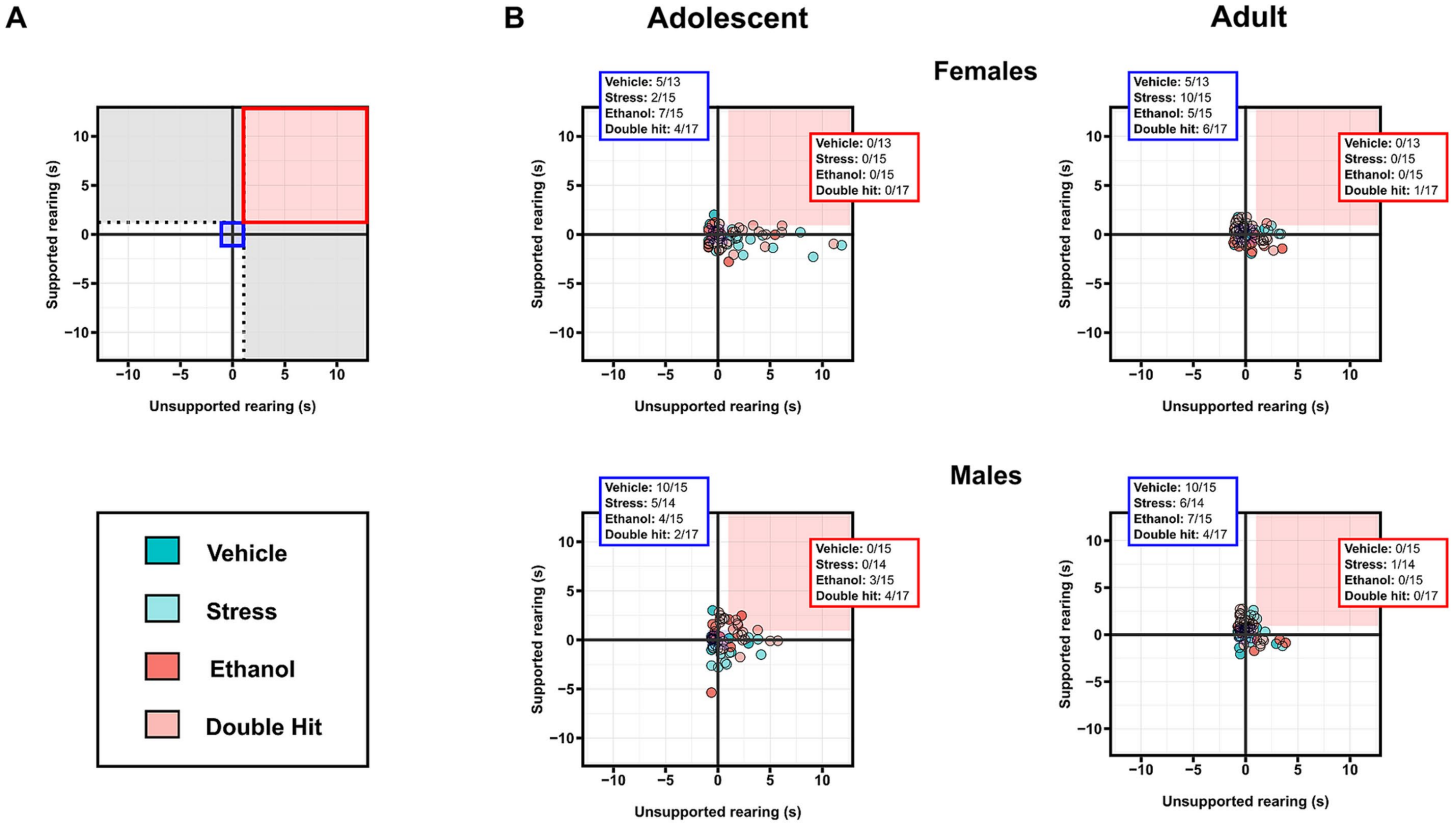
Prenatal alcohol exposure and its combination with juvenile stress increases susceptibility to combined deficits in supported rearing and unsupported rearing in adolescent male offspring. **(A)** Representative two-dimensional matrix. The Vehicle group mean ± one standard deviation is represented by the blue-shaded box in the center of the matrix. The dotted black lines separate individuals who display depressive-like behaviors in only one measure, defined by being outside one standard deviation of the Vehicle group mean in that measure (areas shaded in gray). The blue-shaded box represents the Vehicle group mean ± one standard deviation. The red-shaded box represents individuals who display high supported rearing and high unsupported rearing, defined by being outside one standard deviation of the Vehicle group mean in both measures. Two-dimensional matrices of unsupported rearing x supported rearing for adolescent **(B)** and adult **(C)** offspring from Vehicle (*n* = 28, 13 females, 15 males; 7 litters), Stress (*n* = 29, 15 females, 14 males; 4 litters), Ethanol (*n* = 30, 15 females, 15 males; 4 litters), and Double Hit (*n* = 34, 17 females, 17 males; 5 litters) experimental groups. The proportion of individuals that fall within the red-shaded box is denoted in the red box in the top right corner of the respective matrix. The proportion of individuals that fall within the blue-shaded box is denoted in the blue box in the top left corner of the respective matrix.

**Figure 6:**
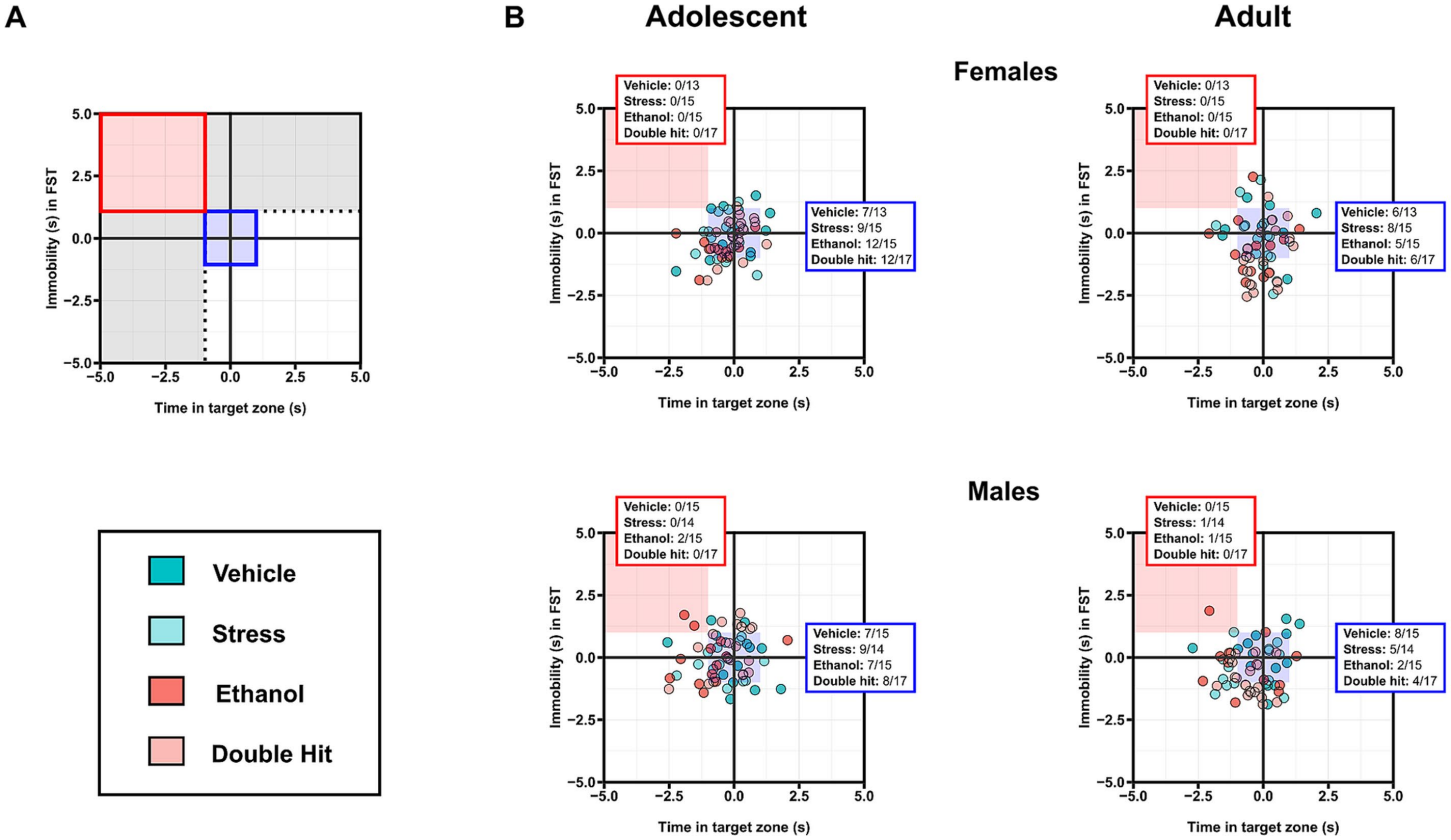
Prenatal alcohol exposure, juvenile stress, and their combination do not increase susceptibility to combined deficits in sociability and immobility in the forced swim test (FST). **(A)** Representative two-dimensional matrix. The Vehicle group mean ± one standard deviation is represented by the blue-shaded box in the center of the matrix. The dotted black lines separate individuals who display depressive-like behaviors in only one measure, defined by being outside one standard deviation of the Vehicle group mean in that measure (areas shaded in gray). The blue-shaded box represents the Vehicle group mean ± one standard deviation. The red-shaded box represents individuals who display low sociability and high immobility, defined by being outside one standard deviation of the Vehicle group mean in both measures. Two-dimensional matrices of sociability x immobility in the FST for adolescent **(B)** and adult **(C)** offspring from Vehicle (*n* = 28, 13 females, 15 males; 7 litters), Stress (*n* = 29, 15 females, 14 males; 4 litters), Ethanol (*n* = 30, 15 females, 15 males; 4 litters), and Double Hit (*n* = 34, 17 females, 17 males; 5 litters) experimental groups. The proportion of individuals that fall within the red-shaded box is denoted in the red box in the top left corner of the respective matrix. The proportion of individuals that fall within the blue-shaded box is denoted in the blue box on the right side of the respective matrix.

Finally, we constructed two-dimensional matrices allowing us to investigate variation across two different behavioral tests simultaneously (Park et al., 2014). Specifically, we constructed matrices of horizontal activity tests (distance traveled × thigmotaxis), vertical activity tests (supported rearing x unsupported rearing), and depression tests (U-field x FST) to compare individual performance across multiple measures. To enable comparisons across different measures, raw behavioral measures were standardized by converting them to *z*-scores using the formula $z=\frac{x-\mu }{\sigma }$ , where *x* is the individual’s outcome, *μ* is the mean value for the Vehicle group, and *σ* is one standard deviation from the Vehicle group mean (Park et al., 2014). *Z*-scores were used to construct the two-dimensional matrices, in which *z*-scores of one measure were plotted on the x-axis, while the other measure was plotted on the y-axis. To interpret the two-dimensional matrices (see Figures 4A, 5A, 6A**)**. Briefly, the dotted black lines separate individuals who display a “deficit” in only one measure, defined by being outside one standard deviation of the Vehicle mean in that respective measure (area shaded in gray) (Park et al., 2014). The solid red box separates individuals who display a “deficit” in both measures, defined by being outside one standard deviation of the Vehicle mean in both measures. The proportion of individuals from each group that fall within the red box was used as a measure of susceptibility to behavioral deficits in the given combination (high distance, low thigmotaxis in Figure 4; high supported rearing, high unsupported rearing in Figure 5; low sociability, high immobility in Figure 6) following early, acute PAE, juvenile SUMS, and their combination.

## 3 Results

### 3.1 Blood alcohol and hair corticosterone outcomes

Following administration of 2 mL/100 g of bodyweight of 31.5% v/v ethanol on GD 7.5, BAC reached a peak of 673.43 ± 133.9 mg/dL at 60-min post gavage (Supplementary Figure S1A), comparable to BAC observed in previous studies of binge-like exposure (Carson and Pruett, 1996; Chang et al., 2019). Furthermore, hair CORT analyses revealed significant stress (*p* < 0.01, *n_p_*^2^ = 0.172; Supplementary Figure S1B) and ethanol x stress effects (*p* < 0.001, *n_p_*^2^ = 0.245; Supplementary Figure S1B) among male offspring, with no differences among female offspring. Following *post hoc* analyses, Ethanol males were found to have significantly higher life-time CORT concentration compared to all other experimental groups.

### 3.2 Early, acute PAE leads to hyperactive behaviors in juvenile offspring

We predicted that juvenile Ethanol offspring would exhibit increased activity behaviors compared to Vehicle mice for all four activity measures. Indeed, Ethanol mice traveled significantly farther (Table 1; *p* < 0.001; η*_p_*^2^ = 0.123) and spent significantly more time rearing (Table 1; *p* < 0.05; η*_p_*^2^ = 0.039), specifically more time in supported rearing (Table 1; *p* < 0.05; η*_p_*^2^ = 0.035) compared to Vehicle offspring. Contrary to our predictions, we observed no differences in thigmotaxis, and the time spent in unsupported rearing between Ethanol and Vehicle mice (Table 1). Additionally, there were no significant sex or ethanol x sex interaction effects for any measures of activity. These findings suggest that early, acute PAE leads to hyperactivity in multiple, but not all, activity measures in juvenile offspring.

**Table 1:** Behavioral outcomes for juvenile (postnatal day 22–26) Vehicle and Ethanol offspring.

Outcome	Vehicle *N* = 57	Ethanol *N* = 64	Ethanol *p* (*η _p_*^2^)	Sex *p* (η _p_^2^)	Ethanol × Sex *p* (η_p_^2^)
Distance traveled (m)	8.79 ± 1.86	10.30 ± 2.11	**<0.001 (0.123)**	0.657 (0.002)	0.162 (0.017)
Thigmotaxis	0.884 ± 0.0577	0.902 ± 0.0531	0.073 (0.027)	0.221 (0.013)	0.133 (0.019)
Total rearing (s)	62.5 ± 13.9	68.4 ± 15.3	**<0.05 (0.039)**	0.987 (0.000)	0.615 (0.002)
Supported rearing (s)	57.4 ± 12.4	62.2 ± 13.4	**<0.05 (0.035)**	0.939 (0.000)	0.249 (0.011)
Unsupported rearing (s)	5.14 ± 4.80	6.13 ± 8.05	0.424 (0.005)	0.913 (0.000)	0.261 (0.011)
Time in target zone (s)	133.6 ± 102.3	155.5 ± 90.0	0.214 (0.013)	0.889 (0.000)	0.502 (0.004)
Immobility (s) in FST	67.3 ± 57.4	47.3 ± 50.5	**<0.05 (0.036)**	**<0.01 (0.064)**	0.887 (0.000)

Behavioral measures for experimental groups are reported as mean ± standard deviation. Results from two-way ANOVAs with ethanol and sex as main effects and treatment × sex as an interaction effect are reported with significant values (*p* < 0.05) bolded.

We predicted that early, acute PAE would lead to less social behavior and that Ethanol offspring would, therefore, spend less time in the target zone than Vehicle mice. However, time spent in the target zone was similar for juvenile Ethanol and Vehicle mice, and there were no significant sex or interaction effects as determined using a two-way ANOVA (Table 1). In the FST, psychomotor withdrawal was measured as the amount of time spent immobile while in the water. We predicted that early, acute PAE would lead to increased psychomotor withdrawal and that Ethanol mice would, therefore, spend more time immobile in the FST than Vehicle offspring. Surprisingly, Ethanol mice spent significantly *less* time immobile than Vehicle offspring (Table 1; *p* < 0.05; η*_p_*^2^ = 0.036). Additionally, there was a significant sex effect with females exhibiting increased immobility compared to males, regardless of treatment. Together, these results suggest that early, acute PAE does not induce depressive-like behaviors in juvenile offspring.

### 3.3 Early, acute PAE and juvenile SUMS elicit increased horizontal activity behaviors in adolescent and adult offspring

As with our juvenile data, we predicted that early, acute PAE, juvenile SUMS, and their combination would alter horizontal activities. Specifically, we expected Ethanol, Stress, and Double Hit mice to travel farther as well as to exhibit less preference for the perimeter resulting in decreased thigmotaxis compared to Vehicle offspring. Results from our two-way ANOVAs indicate that stress significantly increased distance traveled in adolescent (*p* < 0.001; η*_p_*^2^ = 0.120) and to a lesser extent, adult (*p* < 0.05; η*_p_*^2^ = 0.082) female mice. Overall, female offspring in both the Stress and Double Hit groups exhibited increased distance traveled compared to Vehicle and Ethanol mice, indicating that stress leads to increased distance traveled, regardless of PAE (Figure 1B). A similar stress effect was observed for male adolescent (*p* < 0.01; η*_p_*^2^ = 0.172) and adult mice (*p* < 0.01; η*_p_*^2^ = 0.157). However, in contrast to our female data, adolescent and adult males in the Double Hit group show the greatest distance traveled as determined by a significant ethanol x stress interaction effect and Tukey’s *post hoc* analyses (Figure 1B). Together, these data suggest that juvenile SUMS alone increases distance traveled, regardless of sex, but that the double hit of early, acute PAE and juvenile SUMS exacerbates this hyperactive behavior in male offspring only.

Beyond assessing differences in group means for these two tests of horizontal activity, we also investigated the proportion of individuals with a deficit in each behavior measure across age. In females, 40.0% (6/15; RR = 5.20), 26.7% (4/15; RR = 3.47), and 41.2% (7/17; RR = 5.35) of Stress, Ethanol, and Double Hit adolescent offspring displayed high distance traveled compared to 7.7% (1/13) Vehicle adolescent offspring (Figure 1B). Furthermore, 33.3% (5/15; RR = 3.47), 13.3% (2/15; RR = 1.73), and 47.1% (8/17; RR = 6.12) of adult females in these same groups, respectively, exhibited increased distance traveled (Figure 1B). Interestingly, upon closer inspection, those individuals that exhibited high distance traveled as adults were largely not the same individuals as adolescents, suggesting that increased distance traveled may be variable across the lifespan in females. Among the males, only Double Hit offspring exhibited high distance traveled at adolescent (41.2%; 7/17; RR = 6.17) and adult (52.9%; 9/17; RR = 7.94) timepoints (Figure 1B). Contrary to the females, 35.3% (6/17) of male Double Hit offspring displayed increased distance traveled at both ages, suggesting that this phenotype may persist across the lifespan in males.

Thigmotaxis was similarly affected in males and females. At the adolescent timepoint, there was a significant stress effect for thigmotactic behaviors in males (*p* < 0.05; η*_p_*^2^ = 0.109) and females (*p* < 0.001; η*_p_*^2^ = 0.407) such that offspring subjected to juvenile SUMS displayed decreased thigmotaxis, or less preference for the perimeter (Figure 1C). Partial eta squared results indicate that the effect of juvenile SUMS on adolescent females is particularly strong. Early, acute PAE did not affect thigmotaxis in adolescent offspring. In adult mice, there was no significant difference in preference for the perimeter across experimental groups (Figure 1C). These data suggest that stress, regardless of PAE leads to decreased thigmotaxis in adolescent offspring, with the strongest effects observed in females. Notably, 73.3% (11/15; RR = 9.53) and 70.6% (12/17; RR = 9.18) female adolescent offspring in the Stress and Double Hit groups exhibited decreased thigmotaxis compared to 7.7% (1/13) Vehicle offspring (Figure 1C). At the adult timepoint, neither females nor males in the Ethanol, Stress or Double Hit groups displayed differences in thigmotaxis compared to Vehicle offspring suggesting that adolescent Stress and Double Hit females may be particularly susceptible to a low thigmotactic phenotype.

We then investigated the intersection of high distance traveled and low thigmotaxis outcomes. Consistent with our predictions, adolescent females displayed notable differences in this intersection of horizontal activities. Specifically, 60.0% (9/15), 20.0% (3/15), and 70.6% (12/17) of Stress, Ethanol, and Double Hit adolescent female offspring respectively, displayed both high distance traveled and low thigmotaxis compared to 0.0% (0/13) of Vehicle adolescent female offspring (Figure 4B). These results suggest that juvenile SUMS is a strong predictor for developing hyperactive horizontal behaviors in adolescent female mice and that when combined with early, acute PAE, the susceptibility for these outcomes is even greater. The prevalence of combined increased distance traveled and low thigmotaxis in adult female Stress, Ethanol and Double Hit groups is markedly reduced compared to that observed at the adolescent timepoint (Figure 4B). Interestingly, of the adult females that display this combination, eight also displayed simultaneous increased distance traveled and low thigmotaxis as adolescents, suggesting that for some females, the effects of early, acute PAE and juvenile SUMS can persist to adulthood. No notable differences in percentage of individuals that demonstrated both hyperactive behaviors were observed in males across experimental groups at adolescent or adult timepoints (Figure 4B). Collectively, these findings suggest that while both male and female treatment groups displayed increased measures of horizontal activity as adolescents, the manifestation of this behavior differs between sexes.

### 3.4 Early, acute PAE and juvenile SUMS elicit increased vertical activity behaviors in adolescent and adult offspring

As with our juvenile mice, we predicted that early, acute PAE, juvenile SUMS, and their combination would increase vertical activities. For overall rearing, we found a significant ethanol effect for adolescent males (*p* < 0.001; η*_p_*^2^ = 0.276) as well as a significant ethanol × stress interaction (*p* < 0.05; η*_p_*^2^ = 0.066) (Figure 3A). In adult mice, individuals exposed to juvenile SUMS displayed increased rearing overall in both sexes, suggesting that early, acute PAE increased overall rearing exclusively in adolescent males, and that juvenile SUMS increases overall rearing behavior in both female and male adult mice.

Supporting the observed ethanol x stress interaction effect in adolescent males, 41.2% (7/17; RR = 6.18) of adolescent Double Hit males exhibited abnormally high total rearing (Figure 3A), suggesting that adolescent males may be particularly susceptible to increased rearing following the double hit of PAE and stress. A similar finding of 23.5% (4/17; RR = 3.06) of adult Double Hit female offspring displayed high total rearing (Figure 3A), suggesting that age and sex may play a role in the development of this phenotype.

When distinguishing supported rearing from total rearing, we found a significant ethanol effect for adolescent males (*p* < 0.001; η_p_^2^ = 0.211), with individuals exposed to early, acute PAE, regardless of stress, displaying increased time in supported rearing postures (Figure 3B). In adult mice, individuals exposed to juvenile SUMS, regardless of PAE, exhibited increased supported rearing as determined by a significant stress effect for both females (*p* < 0.01; η*_p_*^2^ = 0.169) and males (*p* < 0.05; η*_p_*^2^ = 0.098) (Figure 3B). Moreover, 23.5% (4/17; RR = 3.53) adult Double Hit male offspring had high supported rearing (Figure 3B). These findings suggest that early, acute PAE increases supported rearing for adolescent males only, and that juvenile SUMS increases supported rearing behavior in both male and female adult mice.

Similar to supported rearing outcomes, early, acute PAE led to significantly increased unsupported rearing (*p* < 0.05; η*_p_*^2^ = 0.093) in adolescent males only. However, juvenile SUMS significantly increased unsupported rearing in both female (*p* < 0.001; η*_p_*^2^ = 0.266) and male (*p* < 0.01; η*_p_*^2^ = 0.145) adolescent mice (Figure 3C). The effect of juvenile SUMS was particularly strong in adolescent female offspring with markedly high relative risk of displaying unsupported rearing. Specifically, 66.7% (10/15; RR = 8.67) and 52.9% (9/17; RR = 6.89) female adolescent Stress and Double Hit offspring, respectively, had a high unsupported rearing phenotype (Figure 3C). The effect of juvenile SUMS in females appears to lessen by adulthood as only 17.6% (3/17; RR = 2.29) of Stress adult females and 33.3% (5/15; RR = 4.33) of Double Hit adult females exhibit high unsupported rearing. Together, our findings suggest that juvenile SUMS elicits a greater response for unsupported rearing than early, acute PAE, and that the largest response is found in adolescent females.

Two-dimensional matrix analyses of the intersection of both supported and unsupported rearing indicate that only adolescent males exhibit an increased susceptibility to developing the combination of these behaviors following early, acute PAE and juvenile SUMS. Specifically, 20.0% (3/15) and 23.5% (4/17) of male adolescent Ethanol and Double Hit offspring, respectively, exhibited hyperactivity in both supported and unsupported rearing, compared to 0% of Vehicle (0/15) and Stress (0/14) offspring (Figure 5B). By the adult timepoint, this increased prevalence in combined behaviors in male Ethanol and Double Hit offspring was no longer observed (Figure 5B). The prevalence of combined increased supported and unsupported rearing was very low for female adolescent and adult offspring and similar across experimental groups (Figure 5B). However, these visualizations demonstrate marked variation in unsupported rearing outcomes for adolescent females (Figure 5B). That is, a large majority of adolescent females in the Stress and Double Hit groups exhibit high unsupported rearing compared to Vehicle mice. Together, these findings suggest that the differential outcomes in rearing behavior are complex and are influenced by age, sex, and insult.

### 3.5 Early, acute PAE and juvenile SUMS do not elicit depressive-like behaviors in adolescent or adult offspring

We predicted that Ethanol and Stress offspring would exhibit increased depressive-like behaviors compared to Vehicle mice, and that these behaviors would be exacerbated in the Double Hit mice at the adolescent and adult timepoints. Contrary to our predictions, sociability as measured by time spent in the target zone in the U-field was similar across experimental groups for both adolescent and adult mice (Figure 2B). Similarly, we did not observe a significant increase in time spent immobile in the FST in our Ethanol, Stress, or Double Hit mice compared to Vehicle offspring at the adolescent or adult timepoints (Figure 2C). Consistent with the behavior observed in our juvenile Ethanol mice, both male (*p* < 0.05; η*_p_*^2^ = 0.076) and female (*p* < 0.05; η*_p_*^2^ = 0.071) adult Ethanol offspring spent *less* time immobile than Vehicle mice; a finding that was opposite to our prediction (Figure 2C). Thus, these findings indicate that early, acute PAE and juvenile SUMS, independently and in combination, do not lead to depressive-like behaviors in adolescent and adult offspring.

To compare the proportion of individuals from each experimental group that demonstrated both decreased sociability and increased immobility, we constructed two-dimensional matrices using *z*-scores as described above (Figure 6A). While our analyses of group means for both the sociability and FST behaviors did not reveal a significant difference in depressive-like behaviors across experimental groups, we expected that a greater proportion of Ethanol and Stress adolescent and adult offspring would fall within this “depressed” category compared to Vehicle mice, and that the Double Hit group would have the greatest proportion of mice in this category. However, no notable differences in prevalence among experimental groups were observed in adolescent or adult mice (Figure 6B). These findings support our results from comparisons of group means for each test individually and suggest that early, acute PAE, juvenile SUMS, and their combination do not increase offspring susceptibility to developing these depressive-like behaviors as adolescents or adults.

## 4 Discussion

Previous studies have demonstrated a clear connection between chronic PAE, traumatic juvenile stress, and their combination with adverse behavioral outcomes such as hyperactivity and depression (Alberry et al., 2021; Alberry and Singh, 2016; Comeau et al., 2015; Hellemans et al., 2008, 2010; Kambeitz et al., 2019; Lam et al., 2018a). Given the strong link between these early-life stressors and behavior, it is reasonable to predict that less aggressive forms of these insults such as early, acute PAE and juvenile SUMS will likewise affect hyperactive and depressive-like phenotypes, albeit to a lesser extent. However, despite evidence that many children are likely exposed to alcohol *in utero* before the pregnancy is detected (Canadian Centre on Substance Use and Addiction, 2019; Legault et al., 2021), and that exposure to relatively minor, unpredictable stressors in childhood are common (Craig et al., 2018; Wozniak et al., 2019), the effects of these early life insults on hyperactive and depressive-like behaviors have not been explored. Our study aimed to address this gap in knowledge, incorporating careful inspection of potential sex and age effects, variation in behaviors, and the interplay of outcomes. Our results indicate that early, acute PAE, juvenile SUMS and their combination can indeed impact offspring behavior, but importantly, these effects are not simply scaled-down versions of outcomes reported following more severe insults. Instead, we found that some outcomes mirror those described following chronic PAE and severe juvenile stress, while others appear to be unique to the effects of these less aggressive insults. Below, we interpret and discuss our collective findings.

Given the established link between the early life insults of chronic PAE and severe juvenile stress, and later-life development of depression, we were surprised to find no evidence of depressive-like behaviors in mice following early, acute PAE and juvenile SUMS. Due to the milder nature of the PAE dosing and stress exposure used in this study, we did predict a less severe phenotype than that reported in the literature. However, based on our assays, even when mice were exposed to both early, acute PAE and juvenile SUMS, they showed no evidence of depressive-like behaviors. Indeed, the only statistically significant difference between our experimental and control offspring was a moderate decrease in immobility in the FST for adult male and female mice following ethanol exposure alone. The FST as used in this study, and as established in the field, is a measure of psychomotor withdrawal, and assesses a despair-like phenotype (Can et al., 2012; Planchez et al., 2019; Porsolt et al., 1977). Under this definition, our findings could be interpreted to suggest that early, acute PAE actually *decreases* (though modestly) depressive-like behaviors in adult mice. However, some researchers have suggested that immobility in the FST may be an adaptive response that employs energy-conserving behavior and increases the chance of survival, and as such, the decreased immobility observed in our ethanol-exposed mice could represent a behavioral deficit (Anyan and Amir, 2018; Binik and Sullivan, 1983; Borsini and Meli, 1988; Bruner and Vargas, 1994; Calil and Marcondes, 2006; Commons et al., 2017; Lam et al., 2018b; Nishimura et al., 1988; Schechter and Chance, 1979; West, 1990). Another potential explanation for our findings is that the decreased immobility observed in mice following early, acute PAE is representative of a hyperactive phenotype; a behavior supported by other measures used in this study, and reported previously (Bogdanova et al., 2013; Huang and Huang, 2012). In addition to the FST, we assessed depressive-like behavior using the U-field test for sociability, which in contrast to our predictions, showed no effects of any of the insults on the adolescent or adult mice. Thus, our collective findings indicate that early, acute PAE and juvenile SUMS did not affect depressive-like behaviors. Based on these unexpected results, it would be reasonable to question whether the PAE dosing and juvenile SUMS protocol used here was simply insufficient to elicit a depressive-like phenotype. That said, our measures of hyperactivity, described below, suggest these insults were indeed sufficient to elicit behavioral effects, just not a depressive-like phenotype.

Contrary to our findings for depressive-like behaviors, early, acute PAE and juvenile SUMS alone and in combination caused a hyperactive phenotype in our mice. Specifically, early, acute PAE alone led to hyperactivity in adolescent male mice for supported and unsupported rearing behaviors (Figures 2B,C). Additionally, adolescent and adult male mice subjected to the combination of early, acute PAE and juvenile SUMS displayed increased distance traveled in the U-field (Figure 1B). These outcomes are consistent with those from studies that explored the effects of chronic PAE, wherein susceptibility to developing hyperactive behaviors is highest for males (Cheng et al., 2018; Lam et al., 2018a; Osterlund Oltmanns et al., 2022; Rouzer et al., 2017). In fact, early, acute PAE seemingly had no effect on female activity measures. While we predicted that males would show an exaggerated hyperactive-like behavior following PAE compared to females, we expected to see some effect, as previous studies of chronic PAE have reported hyperactivity in female offspring as well (Hellemans et al., 2008; Ieraci and Herrera, 2020; Sanchez Vega et al., 2013). It is possible that females have a higher threshold for PAE than males in terms of activity outcomes. Ultimately, our findings raise potential clinical concerns as they indicate that even early, acute exposure to alcohol *in utero* can lead to hyperactive behaviors in males.

While early, acute PAE led to hyperactive behaviors in male mice, juvenile SUMS had the most profound effect on activity in both males and females. Following juvenile SUMS, adolescent mice displayed increased distance traveled (Figure 1B), decreased thigmotaxis (Figure 1C), and increased unsupported rearing behavior (Figure 3C); outcomes that attenuated with age. These findings are consistent with reports of previous studies that found increased hyperactive behaviors following severe juvenile stress (García-Díaz et al., 2007; Sequeira-Cordero et al., 2019; Sturman et al., 2021; Torres Muñoz and Franklin, 2022; Ueno et al., 2018). However, based on evidence from studies of chronic PAE and severe juvenile stress, we expected that the greatest measures of hyperactivity would be observed in mice subjected to both early, acute PAE and juvenile SUMS (Hellemans et al., 2008, 2010; Lam et al., 2018a), yet the addition of early, acute PAE had a relatively minor effect on activity outcomes.

The similarity in hyperactive-like behavioral outcomes following juvenile SUMS in both males and females suggests a lack of sex effects, in contrast to findings from previous studies using a severe stress model (Bake et al., 2021; Dunčko et al., 2001; Spivey et al., 2009). However, when one assesses the relative risk, females exhibit a higher risk of hyperactive behavior following juvenile SUMS compared to males, particularly for measures of thigmotaxis and unsupported rearing. Additionally, careful inspection of the interplay between distance traveled and thigmotaxis (Figure 4B) and between supported and unsupported rearing (Figure 5B)—analyses that account for variation in outcomes—reveals marked differences in the response to stress between sexes. Alarmingly, the majority of adolescent females exposed to juvenile SUMS alone or in combination with early, acute PAE demonstrated both increased distance traveled and decreased thigmotaxis. Such an interplay between supported and unsupported rearing was not observed, but this analysis still highlights the variability in unsupported rearing behaviors among adolescent females. Furthermore, it demonstrates that most of the female mice in the Stress and Double Hit groups exhibited increased unsupported rearing compared to Vehicle offspring. Interestingly, this variation in outcomes was not observed in male mice.

The strong sex-specific effect juvenile SUMS on thigmotaxis and unsupported rearing is of interest as both measures are associated not only with activity but also emotionality. Therefore, our findings may indicate that females exhibit altered emotional behavior in addition to hyperactive phenotypes following juvenile SUMS (Sturman et al., 2018, 2021). Previous work has likewise demonstrated this stress-related effect on thigmotaxis and unsupported rearing, and the authors suggested these results indicated an anxiolytic and pro-exploratory phenotype in females (Sturman et al., 2021). Alternatively, this pro-exploratory phenotype among females following juvenile SUMS may be indicative of risk-associated behaviors (Lindberg et al., 2022). As described below, additional behavioral tests are required to discern between these potential explanations for the behaviors observed in the present study.

Overall, juvenile SUMS led to greater behavioral deficits than early, acute PAE. Given our BAC and CORT results, these findings are somewhat surprising. Our ethanol dose achieved exceptionally high BAC levels (average peak of 673.43 mg/dL), comparable to other studies using an early, acute exposure model and confirming that our protocol elicits a binge-like exposure (Brandon-Warner et al., 2012; Carson and Pruett, 1996; Chang et al., 2019). Despite the high BAC achieved, minimal behavioral changes were observed following Ethanol alone. It is possible that an acute dose of ethanol is insufficient to alter these specific behaviors and/or the specific timing used in our protocol does not relate to altered hyperactive and depressive-like behaviors. Future studies are warranted to compare these behavioral outcomes following various ethanol exposure models. Doing this can help determine a threshold of exposure required to alter behavior and tease apart the relative influence of length and timing of exposure on offspring hyperactive and depressive-like behaviors. Our measures of CORT levels are even more puzzling. Levels of CORT are used as a measure of physiological stress experienced by an individual and have been shown to correlate with behavioral outcomes (Dieterich et al., 2019; Shoji et al., 2024). Therefore, given that the largest behavioral deficits were displayed by offspring following juvenile SUMS, we expected to find increased CORT in these experimental groups. However, we are the first to measure and report that elevated CORT was found only following ethanol exposure and only in male mice. Our measures of CORT were performed using hair samples as this approach provides a lifetime measure of stress and avoids a potential spike associated with the stress of blood collection (Marin et al., 2023). For the purpose of our study, this lifetime measure of CORT is preferable to a transient level at the adult timepoint, but it does not allow us to determine if the lack of elevated CORT following juvenile SUMS is because juvenile SUMS did not elevate CORT or because the elevation in CORT immediately following juvenile SUMS was insufficient to significantly increase lifetime CORT measures (Kloet et al., 2008). To better determine the effect of juvenile SUMS on CORT levels, measures derived from blood samples taken immediately after the stress protocol could be used in future work. Another possible explanation for these results is that the effect of juvenile SUMS is mediated by some factor other than CORT. One such factor may be dopamine, as CORT and dopamine generally have an inverse relationship (Dalvi-Garcia et al., 2021). Indeed, previous studies have reported a positive correlation between dopamine levels and hyperlocomotion following acute and mild stress exposure (Butts et al., 2011; Finlay et al., 1995; Lu et al., 2019; Spielewoy et al., 2000). As this is only a suggestion, further investigation is warranted to determine if dopamine is a mediator in the exposure models used in this study.

The specific measures of hyperactivity and depressive-like behavior used in this study were judiciously considered based on their established use in the field, and crucially, on their ability to be repeated multiple times, on the same mouse, throughout its lifespan. That said, we acknowledge that both the U-field test and FST rely on locomotion, which could conflate our measures of hyperactivity and depression (Strekalova and Steinbusch, 2010). Looking forward, in an effort to verify and expand our findings, future studies that incorporate different behavioral tests are warranted to assess depression as well as overall emotionality. For example, while our assessment of sociability using the U-field test and despair using the FST did not reveal a depressed-like phenotype in our experimental groups, it is possible that another test of depressive-like behavior such as anhedonia, as measured by the sucrose preference test, could uncover an effect of early, acute PAE and/or juvenile SUMS (Planchez et al., 2019). Furthermore, because our findings of decreased thigmotaxis and increased unsupported rearing suggest a possible increase in emotionality following juvenile SUMS in females, we recommend that future studies incorporate more explicit tests for emotionality such as the elevated plus maze or light/dark box (Atrooz et al., 2021). Finally, although phenotypic profiling is a fundamental first step toward understanding the effects of early, acute PAE and juvenile SUMS on behavior, future studies that examine how these early life insults affect the development and function of associated neural processes will be essential for the treatment, and ultimately prevention, of these adverse outcomes.

In summary, our findings have clinical relevance as they indicate that even early, acute PAE and juvenile SUMS can lead to behavioral deficits. As with chronic PAE and severe juvenile stress, these outcomes are sex- and age-specific. However, our findings also highlight key differences between the effects of early, acute PAE and juvenile SUMS versus the more severe early life insults. Most notably, adolescent females, rather than males, appear to be most susceptible to developing hyperactive behaviors following juvenile SUMS. Moreover, our results also underscore the importance of considering variation in outcomes in addition to mean group effects, as these data can provide crucial information on offspring susceptibility of developing behavioral deficits. Ultimately, given the potential prevalence of juvenile stress and early, acute alcohol exposure, our collective findings make clear that further exploration of these early-life insults is essential as they can have long-lasting behavioral implications.

## Data Availability

The raw data supporting the conclusions of this article will be made available by the authors, without undue reservation.

## Footnotes

^1^ https://arriveguidelines.org
