# Supplementary material for: Hyperactivity in male and female mice manifests differently following early, acute prenatal alcohol exposure and mild juvenile stress
Source: Front Behav Neurosci. 2025 Mar 18;19:1501937. doi: 10.3389/fnbeh.2025.1501937 (PMC11958967; doi:10.3389/fnbeh.2025.1501937)
Supplement: Supplementary file 1 [file Data_Sheet_1.pdf]

Supplementary Material

Supplemental Table 1. Summary of litter effects.

**Outcome**	**Vehicle**				**Stress**				**Ethanol**				**Double Hit**			
	**Litter *p***				**Litter *p***				**Litter *p***				**Litter *p***			
	**Adolescent**		**Adult**		**Adolescent**		**Adult**		**Adolescent**		**Adult**		**Adolescent**		**Adult**	
	**Female**	**Male**	**Female**	**Male**	**Female**	**Male**	**Female**	**Male**	**Female**	**Male**	**Female**	**Male**	**Female**	**Male**	**Female**	**Male**
Distance travelled (m)	0.584	0.179	**p<0.05**	0.548	0.121	0.064	**p<0.05**	0.735	**p<0.001**	0.374	0.069	**p<0.01**	0.421	0.805	**p<0.05**	**p<0.05**
Thigmotaxis	0.067	0.729	0.095	0.573	0.179	0.065	0.950	0.921	0.337	0.198	0.969	0.517	0.124	0.298	0.357	0.296
Total rearing (s)	0.386	0.775	0.197	0.831	0.544	0.354	0.456	0.109	**p<0.01**	0.887	0.894	0.609	0.370	0.915	0.331	0.297
Supported rearing (s)	0.208	0.993	0.379	0.478	0.562	0.456	0.431	0.057	**p<0.01**	0.642	0.668	**p<0.05**	0.726	0.291	0.961	0.297
Unsupported rearing (s)	0.182	0.605	0.524	0.320	0.223	0.472	0.638	0.973	0.407	**p<0.05**	0.675	0.053	0.306	0.121	0.295	0.669
Time in target zone (s)	0.174	0.861	0.881	0.654	0.941	0.890	**p<0.05**	0.871	0.074	0.958	0.734	0.208	0.184	0.330	0.602	**p<0.05**
Immobility (s) in FST	0.938	0.165	0.793	**p<0.05**	0.109	0.874	**p<0.05**	0.187	0.971	0.130	0.331	0.539	0.537	0.260	0.655	0.328
