# Supplementary material for: Hyperactivity in male and female mice manifests differently following early, acute prenatal alcohol exposure and mild juvenile stress
Source: Front Behav Neurosci. 2025 Mar 18;19:1501937. doi: 10.3389/fnbeh.2025.1501937 (PMC11958967; doi:10.3389/fnbeh.2025.1501937)
Supplement: Supplementary file 2 [file Data_Sheet_2.pdf]

Supplemental Table 2. Juvenile sub-chronic unpredictable mild stress protocol (SUMS).

**Week 1:**	**Day 1**	**Day 2**	**Day 3**	**Day 4**	**Day 5**	**Day 6**	**Day 7**
	**1200-1600h:** Social stress[1]<br>**1600-1800h:** No bedding[2]	**0830-0840h**: Wet cage[3]<br>**0850-0900h:** Wet cage<br>**1200-1500h:** Lights off[4]	**0930-1330h:** Damp bedding[5]<br>**1400-1600h:** Social stress<br>**1630-1830h:** Cage tilt[6]	**1300-1700h:** Cage tilt	**1000-1400h:** Social stress<br>**1400-1600h:** Social stress	**1130-1530h:** No bedding<br>**1530-1730h:** Damp bedding	**0930-1230h:** Succession of 4 light/dark every 30 min[7]

**Week 2:**	**Day 8**	**Day 9**	**Day 10**	**Day 11**	**Day 12**	**Day 13**	**Day 14**
	**1000-1400h:** Social stress	**1200-1400h:** Cage tilt	**0930-1430h:** Succession of 4 light/dark every 30 min<br>**1500-1600h:** Social stress	**1300-1500h:** No bedding<br>**1500-1900h:** Damp bedding	**0800-0900h:** Social stress<br>**1000-1400h:** Cage tilt<br>**1500-1900h:** Social stress	**1130-1330h:** Lights off<br>**1330-1530h:** Social stress	**2030-2330h:** Succession of 4 light/dark every 30 min

**Week 3:**	**Day 15**	**Day 16**	**Day 17**	**Day 18**	**Day 19**	**Day 20**	**Day 21**
	Repeat week 1 schedule.						

**Week 4:**	**Day 22**	**Day 23**	**Day 24**	**Day 25**	**Day 26**	**Day 27**	**Day 28**
	Repeat week 2 schedule.						

[1] Introduction to the (empty) cage of another mouse
[2] Removal of bedding from cage
[3] Empty cage filled with 24°C water to a depth of 1 cm for 10 minutes
[4] Lights are turned off during the light cycle
[5] Bedding is dampened with water
[6] Cage is tilted at a 45° angle
[7] Lights are switched on/off every 30 minutes
