# Supplementary material for: Hyperactivity in male and female mice manifests differently following early, acute prenatal alcohol exposure and mild juvenile stress
Source: Front Behav Neurosci. 2025 Mar 18;19:1501937. doi: 10.3389/fnbeh.2025.1501937 (PMC11958967; doi:10.3389/fnbeh.2025.1501937)
Supplement: Supplementary file 3 [file Data_Sheet_3.pdf]

Supplemental Table 3. Comprehensive summary of behavioural outcomes following prenatal alcohol exposure (PAE) and juvenile sub-chronic unpredictable mild stress (SUMS) in offspring.

**Outcome**	**Vehicle**		**PAE**		**Age *p*** (Within)	**Ethanol *p*** (Between)	**Stress *p*** (Between)	**Sex *p*** (Between)	**Two-way Interactions**	**Three-way Interactions**	**Four-way Interaction**
	**Control** N=28	**Stress** N=29	**Control** N=30	**Stress** N=34							
Distance travelled (m)	14.3 ± 2.74	15.7 ± 3.80	14.1 ± 3.89	17.5 ± 4.01	**p<0.001**	0.055	**p<0.001**	**p<0.001**	**Ethanol x Stress p<0.05**	**Ethanol x Stress x Sex p<0.01**	0.284
Thigmotaxis	0.832 ± 0.0853	0.775 ± 0.074	0.826 ± 0.076	0.770 ± 0.125	0.173	0.633	**p<0.001**	0.193	**Stress x Age p<0.05**	n/a	0.081
Supported rearing (s)	63.0 ± 14.6	61.0 ± 10.9	62.5 ± 17.9	69.2 ± 14.7	**p<0.001**	**p<0.05**	0.250	0.494	**Ethanol x Stress p<0.05**<br>**Ethanol x Sex p<0.01**<br>**Ethanol x Age p<0.01**<br>**Stress x Age p<0.001**	n/a	0.293
Unsupported rearing (s)	7.06 ± 9.71	15.5 ± 13.5	10.7 ± 13.1	14.0 ± 10.5	**p<0.001**	0.488	**p<0.001**	0.808	**Stress x Age p<0.05**	n/a	0.246
Time in target zone (s)	178 ± 65.9	153 ± 49.7	140 ± 58.5	171 ± 44.4	**p<0.05**	0.214	0.666	0.430	**Ethanol x Stress p<0.001**	n/a	0.557
Immobility (s) in FST	125 ± 62.8	109 ± 55.0	99.3 ± 55.2	104 ± 62.3	**p<0.05**	0.090	0.490	0.176	**Ethanol x Age p<0.01**<br>**Stress x Age p<0.05**	n/a	0.799

Note: Behavioural measures for experimental groups are reported as mean ± standard deviation. Results from four-way mixed ANOVAs with age, ethanol, stress, and sex as main effects and interaction effects are reported with significant values bolded. n/a indicates no significant interactions.
