# Supplementary material for: Hyperactivity in male and female mice manifests differently following early, acute prenatal alcohol exposure and mild juvenile stress
Source: Front Behav Neurosci. 2025 Mar 18;19:1501937. doi: 10.3389/fnbeh.2025.1501937 (PMC11958967; doi:10.3389/fnbeh.2025.1501937)
Supplement: Supplementary file 4 [file Data_Sheet_4.pdf]

Supplemental Table 4. Behavioural outcomes following prenatal alcohol exposure (PAE) and juvenile sub-chronic unpredictable mild stress (SUMS) in adolescent (postnatal day 57-61) offspring.

**Outcome**	**Vehicle**		**PAE**		**Ethanol *p* (η_p^2)**	**Stress *p* (η_p^2)**	**Sex *p* (η_p^2)**	**Two-way Interactions**	**Three-way Interaction**
	**Control** N=28	**Stress** N=29	**Control** N=30	**Stress** N=34					
Distance travelled (m)	15.5 ± 2.2	17.4 ± 3.4	15.7 ± 3.8	19.1 ± 2.7	0.068 (0.029)	**p<0.001 (0.186)**	**p<0.01 (0.241)**	n/a	**p<0.01 (0.263)**
Thigmotaxis	0.857 ± 0.071	0.767 ± 0.089	0.842 ± 0.063	0.765 ± 0.075	0.439 (0.005)	**p<0.001 (0.258)**	0.322 (0.009)	**Stress x Sex p<0.05 (0.056)**	**p<0.05 (0.038)**
Supported rearing (s)	70.8 ± 11.6	59.9 ± 11.2	72.7 ± 16.9	74.0 ± 12.8	**p<0.001 (0.092)**	0.066 (0.030)	0.807 (0.001)	**Ethanol x Stress p<0.05 (0.053)**<br>**Ethanol x Sex p<0.05 (0.056)**	0.748 (0.001)
Unsupported rearing (s)	3.4 ± 4.9	12.1 ± 11.6	5.5 ± 6.2	14.1 ± 10.1	0.159 (0.017)	**p<0.001 (0.209)**	0.947 (0.000)	n/a	0.417 (0.006)
Time in target zone (s)	185 ± 66.4	160 ± 47.4	142 ± 57.6	184 ± 45.1	0.4366 (0.005)	0.2797 **(0.010)**	0.8527 (0.000)	**Ethanol x Stress p<0.01 (0.091)**	0.547 (0.003)
Immobility (s) in FST	122 ± 68.6	108 ± 54.2	101 ± 51.1	131 ± 59.4	0.871 (0.002)	0.385 (0.063)	0.323 (0.080)	**Ethanol x Stress p<0.05 (0.272)**	0.895 (0.002)

Note: Behavioural measures for experimental groups are reported as mean ± standard deviation. Results from three-way ANOVAs with ethanol, stress, and sex as main effects and interaction effects are reported with significant values bolded. n/a indicates no significant interactions.
