# Supplementary material for: Hyperactivity in male and female mice manifests differently following early, acute prenatal alcohol exposure and mild juvenile stress
Source: Front Behav Neurosci. 2025 Mar 18;19:1501937. doi: 10.3389/fnbeh.2025.1501937 (PMC11958967; doi:10.3389/fnbeh.2025.1501937)
Supplement: Supplementary file 5 [file Data_Sheet_5.pdf]

Supplemental Table 5. Behavioural outcomes following prenatal alcohol exposure (PAE) and juvenile sub-chronic unpredictable mild stress (SUMS) in adult (postnatal day 120-124) offspring.

**Outcome**	**Vehicle**		**PAE**		**Ethanol *p* (η_p^2)**	**Stress *p* (η_p^2)**	**Sex *p* (η_p^2)**	**Two-way Interactions**	**Three-way Interaction**
	**Control** N=28	**Stress** N=29	**Control** N=30	**Stress** N=34					
Distance travelled (m)	13.0 ± 2.7	14.0 ± 3.4	12.6 ± 3.4	16.0 ± 4.5	0.149 (0.018)	**p<0.001 (0.101)**	**p<0.01 (0.079)**	n/a	0.055 (0.032)
Thigmotaxis	0.807 ± 0.092	0.783 ± 0.087	0.809 ± 0.085	0.776 ± 0.161	0.880 (0.000)	0.158 (0.018)	0.213 (0.014)	n/a	0.281 (0.010)
Supported rearing (s)	55.2 ± 13.2	62.0 ± 10.7	524 ± 12.4	64.4 ± 15.1	0.975 (0.000)	**p<0.001 (0.127)**	0.234 (0.013)	n/a	0.344 (0.008)
Unsupported rearing (s)	10.7 ± 11.9	18.8 ± 14.6	15.9 ± 16.0	13.8 ± 11.0	0.997 (0.000)	0.273 (0.011)	0.751 (0.001)	**Ethanol x Stress p<0.05 (0.036)**	0.4699 (0.005)
Time in target zone (s)	172 ± 66.0	147 ± 51.8	139 ± 60.3	158 ± 40.2	0.319 (0.009)	0.871 (0.000)	0.150 (0.018)	**Ethanol x Stress p<0.05 (0.040)**	0.905 (0.000)
Immobility (s) in FST	128 ± 57.5	109 ± 56.7	97.4 ± 59.9	78.0 ± 54.0	**p<0.01 (0.073)**	0.068 (0.029)	0.236 (0.012)	n/a	0.678 (0.002)

Note: Behavioural measures for experimental groups are reported as mean ± standard deviation. Results from three-way ANOVAs with ethanol, stress, and sex as main effects and interaction effects are reported with significant values bolded. n/a indicates no significant interactions.
