# Supplementary material for: Hyperactivity in male and female mice manifests differently following early, acute prenatal alcohol exposure and mild juvenile stress
Source: Front Behav Neurosci. 2025 Mar 18;19:1501937. doi: 10.3389/fnbeh.2025.1501937 (PMC11958967; doi:10.3389/fnbeh.2025.1501937)
Supplement: Supplementary file 6 [file Image_1.pdf]

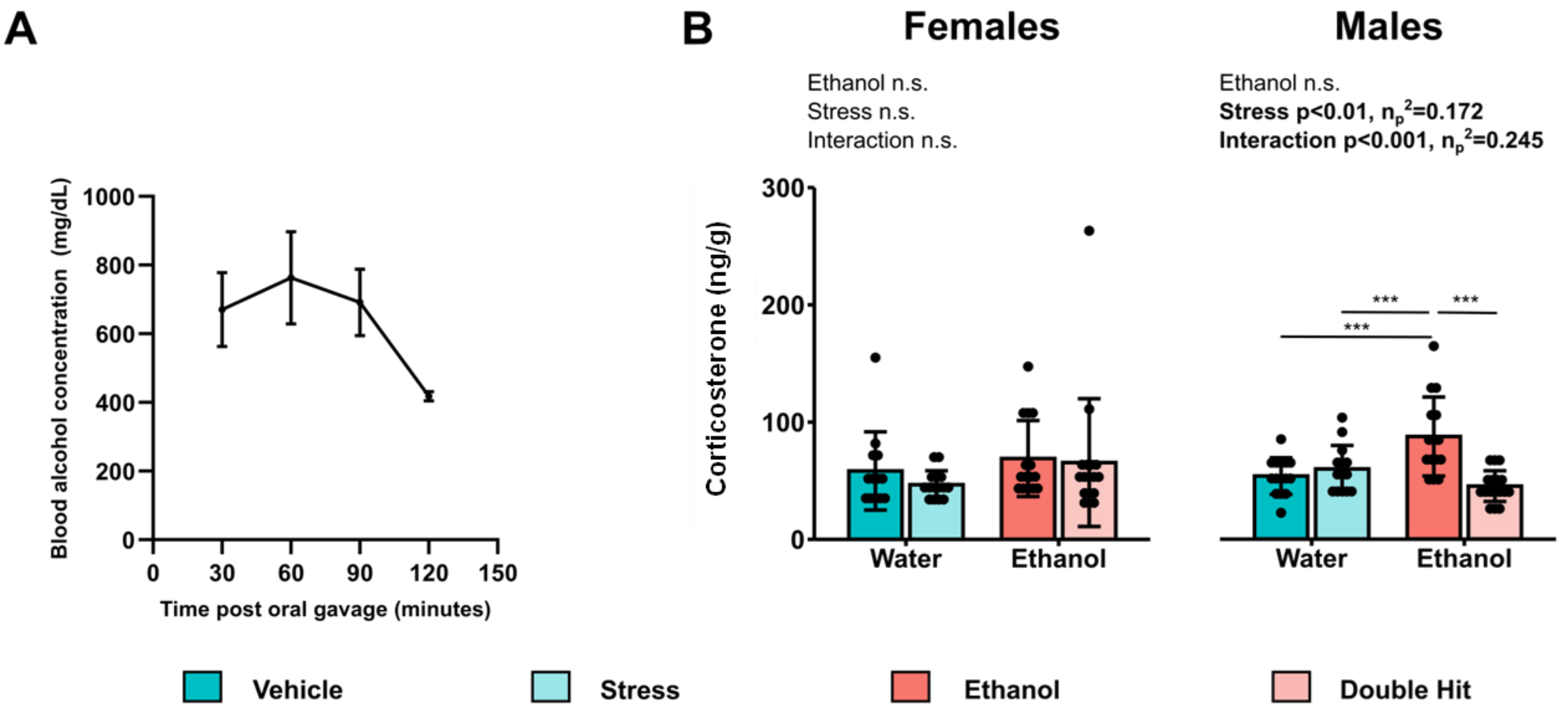

Figure 1. Measurement of blood alcohol concentration (BAC) and hair corticosterone following early, acute prenatal alcohol exposure and juvenile sub-chronic, unpredictable, mild stress respectively. (A) A separate cohort of nulliparous pregnant females (n=4) were administered 31.5% v/v ethanol (2 ml/100 g of bodyweight) on gestational day 7.5 and BAC of plasma samples at 30-, 60-, 90-, and 120-minutes was quantified using the Analox analyzer model GM7 MicroStat (Analox Instruments, Lunenburg, MA). BAC was normalized by subtracting background (water-dosed mice BAC) and plotted against time as mean ± standard deviation. BAC reached a peak of 673.43 ± 133.9 mg/dL at 60-minutes post gavage. **(B)** Following the completion of behavioural tests, hair samples were collected from offspring from Vehicle (n=28, 13 females, 15 males; 7 litters), Stress (n=29, 15 females, 14 males; 4 litters), Ethanol (n=30, 15 females, 15 males; 4 litters), and Double Hit (n=34, 17 females, 17 males; 5 litters) experimental groups and used to measure lifetime corticosterone levels. Mean corticosterone values for each group are represented by bars ± standard deviation. Data were compared using two-way ANOVAs (ethanol, stress), followed by Tukey's *post hoc* analyses where appropriate. ***p<0.001, n.s. = not significant.
